# Improving Dermal Delivery of Rose Bengal by Deformable Lipid Nanovesicles for Topical Treatment of Melanoma

**DOI:** 10.1021/acs.molpharmaceut.1c00468

**Published:** 2021-09-23

**Authors:** Sara Demartis, Giovanna Rassu, Sergio Murgia, Luca Casula, Paolo Giunchedi, Elisabetta Gavini

**Affiliations:** † Department of Chemistry and Pharmacy, University of Sassari, 07100 Sassari, Italy; ‡ Department of Life and Environmental Sciences, University of Cagliari, 09042 Monserrato, Cagliari, Italy; § CSGI, Consorzio Interuniversitario per lo Sviluppo dei Sistemi a Grande Interfase, 50019 Sesto Fiorentino, Florence, Italy

**Keywords:** liposome, nanoparticle, rose bengal, melanoma, skin cancer, dermal delivery

## Abstract

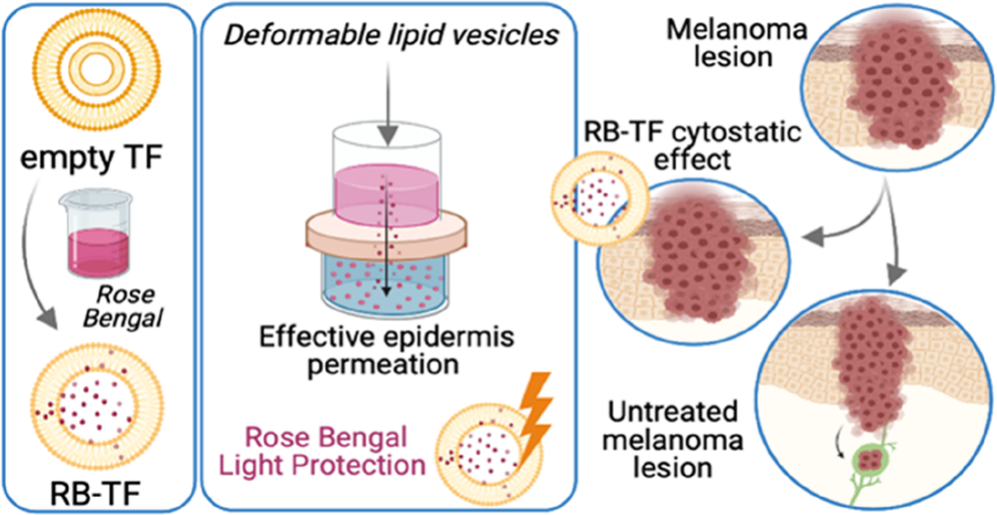

Cutaneous melanoma is one of the most aggressive and metastatic forms of skin cancer. However, current therapeutic options present several limitations, and the annual death rate due to melanoma increases every year. Dermal delivery of nanomedicines can effectively eradicate primary melanoma lesions, avoid the metastatic process, and improve survival. Rose Bengal (RB) is a sono-photosensitizer drug with intrinsic cytotoxicity toward melanoma without external stimuli but the biopharmaceutical profile limits its clinical use. Here, we propose deformable lipid nanovesicles, also known as transfersomes (TF), for the targeted dermal delivery of RB to melanoma lesions to eradicate them in the absence of external stimuli. Considering RB’s poor ability to cross the stratum corneum and its photosensitizer nature, transfersomal carriers were selected simultaneously to enhance RB penetration to the deepest skin layers and protect RB from undesired photodegradation. RB-loaded TF dispersion (RB-TF), prepared by a modified reverse-phase evaporation method, were nanosized with a ζ-potential value below −30 mV. The spectrophotometric and fluorimetric analysis revealed that RB efficiently interacted with the lipid phase. The morphological investigations (transmission electron microscopy and small-angle X-ray scattering) proved that RB intercalated within the phospholipid bilayer of TF originating unilamellar and deformable vesicles, in contrast to the rigid multilamellar unloaded ones. Such outcomes agree with the results of the in vitro permeation study, where the lack of a burst RB permeation peak for RB-TF, observed instead for the free drug, suggests that a significant amount of RB interacted with lipid nanovesicles. Also, RB-TF proved to protect RB from undesired photodegradation over 24 h of direct light exposure. The ex vivo epidermis permeation study proved that RB-TF significantly increased RB’s amount permeating the epidermis compared to the free drug (78.31 vs 38.31%). Finally, the antiproliferative assays on melanoma cells suggested that RB-TF effectively reduced cell growth compared to free RB at the concentrations tested (25 and 50 μM). RB-TF could potentially increase selectivity toward cancer cells. Considering the outcomes of the characterization and cytotoxicity studies performed on RB-TF, we conclude that RB-TF represents a valid potential alternative tool to fight against primary melanoma lesions via dermal delivery in the absence of light.

## 1 Introduction

According to the Italian Association for Cancer Research (AIRC), cutaneous melanoma represents only 5% of skin cancers. Nevertheless, its ability to rapidly metastasize makes melanoma the most aggressive and deadly form of skin cancer. It remains a significant health problem worldwide, with death often occurring due to metastasis.^1^ Statistics predicted that around 106,110 cases of melanoma would be diagnosed in the United States in 2021, and about 7,180 people are expected to die ;^2^ also, the incidence of invasive melanoma in European regions showed an average annual increase of 4.0% in men and 3.0% in women during the 1995–2012 period.^3^ Cutaneous melanoma typically localizes in the epidermis’ bottom layers or deeper in the dermis and originates from melanocytes’ tumor transformation. Depending on the location and stage, the primary cutaneous melanoma can be treated via surgical resection but it cannot be applied in all cases. Current therapeutic approaches, including chemotherapy, radiotherapy, immunotherapy, and photodynamic therapy, have reported two main limitations: adverse effects (usually related to an immune reaction and lack of specificity) and induced resistance to immune–chemotherapeutics or intralesional therapy.^4^ Thus, better therapeutic options are required. For this purpose, primary melanoma lesions can be effectively eradicated via dermal delivery of therapeutics, increasing patient compliance. Indeed, the dermal route offers direct access to melanoma cells avoiding systemic administration and its disadvantages. Considering the complexity of human skin, the challenge in developing an effective dermal delivery system is to bypass the skin barrier and target the drug only to cancer cells.^1,5^

**Figure 1:**
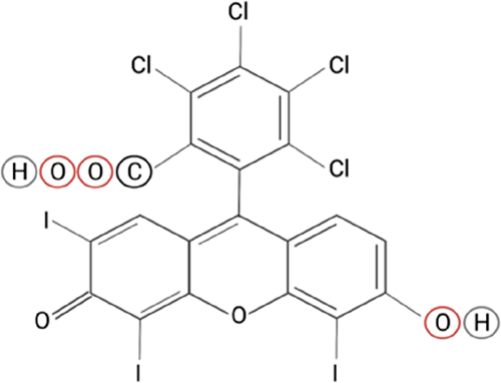
Rose Bengal chemical structure. Created with BioRender.com.

Rose Bengal (RB) (3′,4′,5′,6′-tetrachloro-2,4,5,7-tetraiodofluorescein) is a dye with a xanthenic structure mainly used in ophthalmology as a diagnostic tool^6^ (Figure 1).

Moreover, RB is a sono-photosensitizer drug employed in sono-photodynamic therapy and demonstrated intrinsic cytotoxicity against tumor and microbial cells.^6^ Of relevant importance in this context is RB’s antimelanoma activity in the absence of external stimuli, including light or ultrasounds. In particular, RB was reported to induce death in different cultured melanoma cell lines, probably via necrosis, even if both caspase-dependent and -independent apoptotic pathways were observed.^7,8^ PV-10 is a 10% RB solution currently tested to treat melanoma after intralesional injection. PV-10 acts through a dual mechanism: it first induces chemical ablation of the tumor and a second systemic effect following the onset of a tumor-specific immune response.^9,10^ Therefore, RB can be proposed as a valid alternative for both local and metastatic melanoma.

Despite its potential, RB’s clinical application is limited by its biopharmaceutical profile. RB (employed in the disodium salt form) is an amphiphilic and water-soluble molecule with a molecular weight of 1017.64 g mol^–1^, presenting two negative charges in solution.^6^ For an efficient permeation through the skin layers, a drug first needs to pass the stratum corneum (SC), which is the main barrier against the penetration of external agents. Considering the anionic nature and the high molecular weight, RB would not easily pass the SC and therefore cannot be considered a drug candidate suitable for the cutaneous administration route.^11,12^ In addition, RB suffers from poor cell crossing hampering suitable accumulation in the target cells. Nevertheless, nano-based delivery systems have proved to overcome the RB limits mentioned above efficiently.^6^

Lipid-based nanosystems were widely employed for the dermal delivery of photosensitizer drugs.^13,14^ Among these, transfersomes (TF) are considered the most innovative dermal and transdermal carriers to date.^15^ TF are liposome-like vesicles consisting of a hydrophilic core surrounded by a hydrophobic bilayer but the additional surfactant present in the phospholipid arrangement provides ultra flexibility.^16^ A conventional liposome presents the disadvantage of poor penetration into the viable skin, limiting its therapeutical application to the diseases involving outermost skin layers. Because of their composition, the elasticity of TF increases, making them squeeze and pass through skin pores smaller than their size. Considering that TF tend to avoid dry surroundings, the sufficient force to drive them through the skin is generated by the transdermal hydration gradient, the increasing water concentration gradient from the superficial to the inner skin strata. In conventional liposomes, the rigid structure hampers the migration as they require high energy to deform. On the other hand, TF can deform without losing their integrity, and undesirable leakage and drug loss are avoided. As a result, the drug is efficiently delivered in the deepest region of the skin.^17,18^ Although the mechanism above is considered the primary one operating, other mechanisms can be involved in the transfersomal action.^18,19^

The current work aims to provide a dermal delivery of RB to melanoma lesions via optimizing its biopharmaceutical profile and enhancing RB’s intrinsic antimelanoma activity in the absence of light or ultrasounds. To achieve this goal, RB has been formulated in TF made of phosphatidylcholine (Lipoid S 100), cholesterol, and Span 80. RB-loaded TF were characterized in dimensional properties, ζ-potential, and morphology; in addition, the storage stability and photostability of RB-loaded TF and corresponding blank formulation were determined. Spectrophotometric, fluorimetric, and small-angle X-ray scattering (SAXS) analyses were performed to evaluate the interaction between the dye and the lipid phase of vesicles. Ex vivo epidermis permeation experiment determined TF’s ability to permeate across the epidermal barrier reaching the dermis. Finally, an in vitro toxicity study on melanoma cells (SK-MEL28) was carried out to investigate the new RB delivery system’s antimelanoma efficacy.

## 2 Materials and Methods

### 2.1 Materials

Rose Bengal sodium salt (RB), cholesterol, Span 80, and ethanol were purchased by Sigma-Aldrich (St. Louis, MO). Lipoid S 100 was gifted by Lipoid GmbH (Ludwigshafen, Germany). Acetonitrile and dimethylsulfoxide (DMSO) were acquired from Merck (Darmstadt, Germany). Phosphate-buffered saline (PBS, NaCl 0.138 M; KCl 0.0027 M; pH 7.4; 25 °C) was obtained by Sigma-Aldrich (Milan, Italy). Ultrapure bidistilled water was obtained by a MilliQ R4 system, Millipore (Milan, Italy).

### 2.2 Preparation and Characterization of TF

#### 2.2.1 TF Preparation

A previous preformulation study set the most suitable manufacturing parameters to prepare unloaded and RB-loaded TF (Supporting Information), including the preparative technique, the choice of the organic solvent and the sonication technique, and time.

RB-loaded TF dispersion (RB-TF) was prepared by a slightly modified reverse-phase evaporation method (REV).^20^ The lipid phase consisting of a mixture of 400 mg of Lipoid S 100 (phosphatidylcholine), 75 mg of cholesterol, and 40 μL of Span 80 was dissolved in 7 mL of ethanol at 50 °C and mixed with 10 mL of an aqueous solution (MilliQ water) containing 5 mg of RB. After sonication treatment using a probe sonicator Bioblock Vibracell (Fisher Bioblock Scientific, Illkirch, France) for 30 s at 50% ultrasound (US) amplitude, the resulting dispersion was placed in a dry, round-bottom flask. Ethanol was removed under vacuum (Rotavapor RE111, Büchi Labortechnik AG, Flawil, Switzerland) at 50 °C, and the vesicles were swollen for 1 h at room temperature and sonicated by three probe sonication cycles: each cycle consisted of 10 s of US followed by an interval of 20 s.^21^ Finally, RB-TF dispersion was extruded 10 times through a regenerated cellulose syringe filter (pore size: 0.45 μm, filter size: 25 mm, AlfaTech, Genova, Italy) and stored at 4 °C. The dispersion of corresponding unloaded vesicles (b-TF) was prepared.

#### 2.2.2 Particle Size and ζ-Potential Analysis

Dimensional analyses of RB-TF and b-TF were performed by photon correlation spectroscopy (PCS). A Coulter nanosizer N5 (Beckman-Coulter Inc., Miami, FL) was used to determine the hydrodynamic diameter and the polydispersity index (PDI) to measure the dimensional heterogeneity of the sample. Before each analysis, all samples were diluted adequately with MilliQ water previously filtered (regenerated cellulose syringe filter, pore size: 0.20 μm, filter size: 15 mm, Albet LabScience, Dassel, Germany) to ensure light-scattering intensity within the required range of the instrument (between 5 × 10^4^ and 1 × 10^6^ counts s^–1^). Particle size was calculated in unimodal using the following conditions: fluid refractive index 1.333; temperature 25 °C; viscosity 0.890 cP; angle of measurement 90°; sample time 3.0 ms; and sample run time 300 s. ζ-Potential was measured using the Zetasizer Nano (Malvern Instrument, Worcestershire, United Kingdom) through the M3-PALS (Phase Analysis Light Scattering) technique. Just before the analysis, the formulations were diluted with distilled water.

#### 2.2.3 Drug Content

The RB content in the RB-TF dispersion was evaluated as described here. Thirty microliters of the RB-TF dispersion was diluted up to 2 mL with acetonitrile under magnetic stirring to dissolve the nanovesicles and extract RB. Successively, the RB content was measured with a UV-spectrophotometer (Shimadzu UV-1800, Kyoto, Japan) and calculated referring to the calibration curve prepared in acetonitrile (standard solution in the range of 1–10 mg L^–1^; *y* = 101610959*x* + 0.020676712; *R*^2^ = 0.999). Finally, the drug content (DC%) was determined according to eq 1
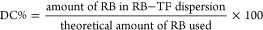
1

#### 2.2.4 Transmission Electron Microscopy (TEM)

RB-TF and b-TF were analyzed through transmission electron microscopy (TEM) to investigate the vesicles’ morphology. A drop of sample and an equal volume of an aqueous 1% phosphotungstic acid solution were adsorbed on the surface of a carbon-coated copper grid (200 mesh). The vesicles were observed after drying at room temperature using a JEOL JEM 1400 Plus (JEOL Ltd., Tokyo, Japan) with an accelerating voltage of 80 kV in the bright-field mode.

#### 2.2.5 Degree of Deformability

**Figure 2:**
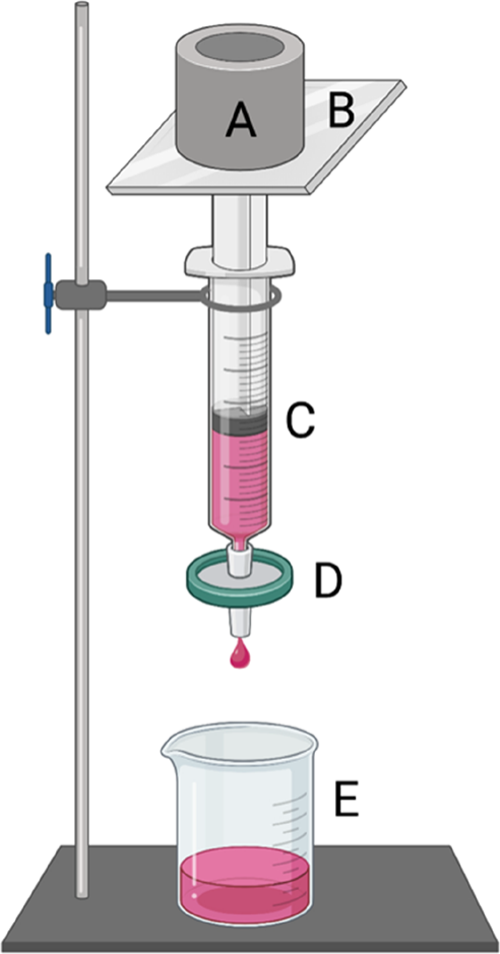
Schematic illustration of the modified extrusion device employed to evaluate the deformability indexes of RB-TF and b-TF: (A) 500 g calibration weight; (B) weight support; (C) syringe; (D) support for the membrane filter (0.2 μm pore size); and (E) collecting beaker. Created with BioRender.com.

Deformability tests of b-TF and RB-TF were performed using a modified extrusion device^22^ illustrated in Figure 2.

The samples (2 mL) were withdrawn with a syringe successively connected to a membrane filter with a pore size of 0.2 μm (regenerated cellulose membrane filter, filter size: 47 mm, Whatman, GE Healthcare Life Sciences, Buckinghamshire, United Kingdom);^23,24^ extrusion process was performed by constantly applying a calibration weight of 500 g on the top of the syringe. Particle size was determined just before and after experimenting; deformability index was measured following eq 2(25)
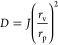
2where *D* is the deformability index of nanovesicles; *J* is the amount of formulation extruded in 5 min; *r*_v_ is the particle size after the experiment; and *r*_p_ is the membrane pore size.

#### 2.2.6 Small-Angle X-ray Scattering (SAXS)

SAXS was recorded with an S3-MICRO SWAXS camera system (HECUS X-ray Systems, Graz, Austria). A GeniX X-ray generator, operating at 1 mA and 50 kV, provided a Cu Kα radiation of 1.542 Å wavelength. The scattered X-rays were detected in the small-angle region by a 1D-PSD-50 M system (Hecus X-ray Systems, Graz, Austria) containing 1024 channels of 54.0 mm width. The working *q*-range (Å) was 0.002 ≤ *q* ≤ 0.06, with *q* = 4π sin(θ)λ^–1^ being the modulus of the scattering wave vector. Glass capillaries of 2 mm were used in experiments while diffraction patterns were recorded for 10 800 s. Scattering from the air was minimized, keeping the camera volume under vacuum during measurements. Silver behenate (CH_3_–(CH_2_)_20_–COOAg), with a *d* spacing value of 58.38 Å was the standard for calibration of the angular scale of the measured intensity. The SAXS pattern of RB-TF was analyzed by GAP (Global Analysis Program), which allows the fitting of bilayer-based structures.^19,26^ Particularly, the membrane thickness (*d*_B_) was defined as 2(*Z*_H_ + 2σ_H_), where *Z*_H_ and σ_H_ are parameters obtained from SAXS curve fitting and, respectively, represent the head group-to-bilayer center distance and the polar head amplitude. The latter parameter was kept fixed at 3 A° as suggested by the software developers.^26^

### 2.3 Evaluation of the Interaction between RB and the Lipid Phase

Studies on absorption (Abs) and emission (Ems) spectra of (i) RB aqueous solution, (ii) RB-TF dispersion, and (iii) RB added in b-TF dispersion were recorded as previously proposed by Chang et al.,^27^. Abs spectra were measured with a UV-Spectrophotometer (Shimadzu UV-1800, Kyoto, Japan); and Ems spectra were recorded with an RF-600 spectrofluorometer (Shimadzu, Kyoto, Japan), exciting each sample at 549 nm. Before each measurement, all samples were diluted with MilliQ water previously filtered (regenerated cellulose syringe filter, pore size: 0.20 μm, filter size: 15 mm, Albet LabScience, Dassel, Germany) up to 7.5 mg L^–1^ RB concentration. Spectra of b-TF dispersion were recorded following the same procedure.

### 2.4 Stability Studies

#### 2.4.1 Storage Stability

b-TF and RB-TF dispersions were stored for 60 days at 4 °C, protected from light. At each predetermined time point (1, 3, 7, 15, 30, 60 days), samples were analyzed in terms of dimensional properties and ζ-potential as described in Section 2.2.2.

#### 2.4.2 Chemical Stability

The chemical stability of RB-TF dispersion was evaluated in terms of RB content over time and compared with the RB aqueous solution after storing samples at 4 °C protected from light. Samples were analyzed at each predetermined time point (1, 3, 7, 15, 30, 60 days), as described in Section 2.2.3. The RB amount was calculated for the RB aqueous solution, referring to the calibration curve prepared in water (standard solution in the range of 1–20 mg L^–1^; *y* = 0.10640119*x* + 0.00531086; *R*^2^ = 0.999).

#### 2.4.3 Photostability

The photostability of RB-TF dispersion was evaluated and compared with the RB aqueous solution. One milliliter of each sample (0.5 mg of RB) was exposed to light in the visible region delivered from an artificial daylight lamp (220 V, 40 W) over 24 h at room temperature. Photodegradation of RB was monitored at each predetermined time point (1, 2, 3, 6, 24 h) in terms of RB content by UV-vis spectrophotometry. The RB content in RB-TF dispersion was determined as described in Section 2.2.3; for RB aqueous solution, the amount of RB was calculated referring to the calibration curve prepared in water as mentioned previously.

### 2.5 In Vitro Release Study

The in vitro release profile of RB-TF dispersion was evaluated across a polycarbonate membrane (0.050 μm pore size, 47 mm diameter, Sigma-Aldrich, St. Louis, MO) using a dissolution apparatus (DT 70, Erweka, Langen, Germany), as previously described by Gavini et al.^28^ The membrane was mounted on the bottom of a cylindrical plastic support consisting of a tube (height = 1.91 cm, diameter = 2.28 cm) connected to a drive shaft of the dissolution apparatus (Supporting Information). The membrane was clamped to the support by a plastic ring, and then 2 mL of the sample (1 mg of RB) was placed on the surface of the membrane. The system was then inserted into the vessel containing 250 mL of PBS as the acceptor medium, keeping the membrane in contact with the surface of the PBS all the time. The system was continuously stirred at 100 rpm and thermostated at 32 °C; it was allowed to equilibrate for 30 min before starting the experiment. The samples of the acceptor medium (3 mL) were collected at each predetermined time point (0.08, 0.17, 0.25, 0.5, 1, 2, 3, 6, 24, 32, 48 h); in the case of RB-TF dispersion, acetonitrile was used to ensure the complete recovery of RB. The RB aqueous solution was also tested as a comparison. The amount of dye in the acceptor medium was measured by UV–vis spectrophotometry and calculated using the calibration curve prepared in acetonitrile for RB-TF dispersion and in water for RB. An equal volume of the fresh medium was immediately replaced after each sampling. The cumulative amount of RB was plotted against time and determined through eq 3
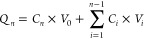
3where *Q_n_* is the cumulative amount of the drug; *C_n_* is the drug concentration of the acceptor medium at each time point; *V*_0_ is the acceptor medium’s volume; *C_i_* is the drug concentration of the sample at each time point; and *V_i_* is the volume of the sample collected at each time point.

### 2.6 Ex Vivo Epidermis Permeation and Retention Study

**Figure 3:**
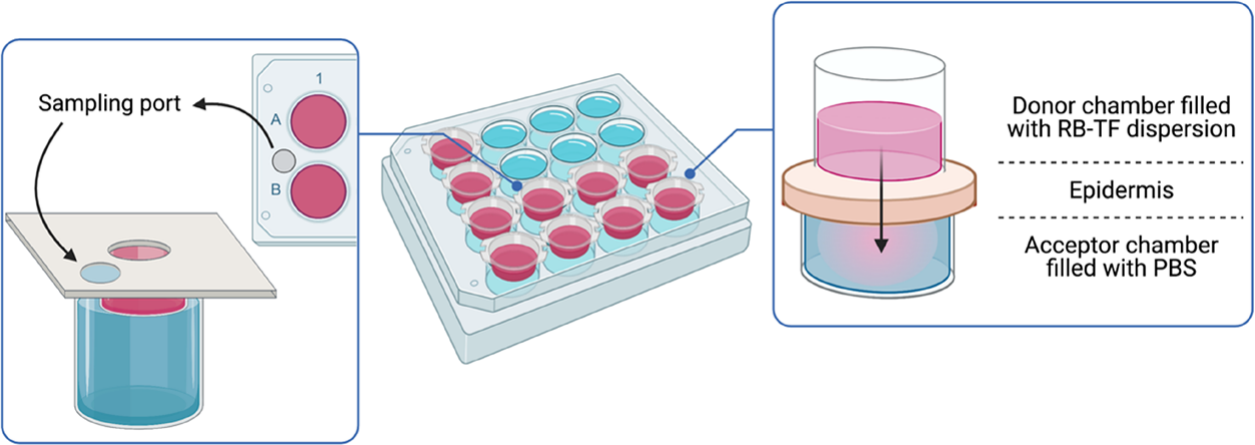
Schematic illustration of the diffusion apparatus (project INCREASE SARDINIA 2016–17, protocol number 31351, University of Sassari) employed to evaluate the ex vivo epidermis permeation profile of RB-TF dispersion and RB aqueous solution. Created with BioRender.com.

Ex vivo permeation of RB-TF dispersion and RB aqueous solution was performed across epidermal membranes excised from porcine ears. Adult pig ears were obtained from a local slaughterhouse (Milia S.r.L, Approval Number CE IT 1856 M (Regulation EC 853/2004)). Epidermal membranes, comprising viable epidermis and stratum corneum, were prepared by the heat separation technique described here:^29^ hairs of ears were removed using a hair clipper; shaved ears were immersed in water at 60 °C for about 2 min; following removal from the water, the epidermis was gently peeled off. The experiment was performed using a 12-multiwell cell culture plates suitability modified^30^ (project INCREASE SARDINIA 2016–17, protocol number 31351, University of Sassari) as illustrated in Figure 3.

Each well had an effective diffusion area of 1 cm^2^. Wells were filled with 5.5 mL of PBS (pH. 7.4) as an acceptor medium, and the epidermis was mounted on the plates with the inner side facing PBS. RB-TF dispersion (0.3 mL, 0.15 mg of RB) was added on the top of the epidermis and the plates were incubated at 32 °C (SKI 4 Shaker Incubator, Argo Lab, Carpi, Italy). The system was allowed to equilibrate for 30 min before starting the experiment. Samples (0.2 mL) were withdrawn at each predetermined time point (0.25, 0.5, 1, 3, 5, 8, 24 h) from a sampling port directly connected to the receptor chamber; successively, the samples were added to 1 mL of DMSO to dissolve both lipids of RB-TF dispersion and skin to extract RB completely. The amount of RB permeated was measured by UV–vis spectrophotometry and calculated using the calibration curve prepared in DMSO (standard solution in the range of 2–12 mg L^–1^; *y* = 87.814*x* – 0.1041, *R*^2^ = 0.998). After each sampling, an equal volume of fresh medium was immediately replaced through the sampling port previously employed to withdraw the sample. The same procedure was applied to evaluate the ex vivo permeation profile of the RB aqueous solution. The cumulative amount of RB permeated was plotted against time and determined as described in Section 2.5.

At the end of the permeation experiment, the epidermis was carefully removed from the plate and cut into small pieces, added to 2 mL PBS, and boiled for 10 min; after that, 2 mL of DMSO was added to ensure RB extraction and further boiled for 10 min. The amount of RB retained in the tissue was measured with a UV–vis spectrophotometer and calculated using the DMSO calibration curve.

### 2.7 Cytotoxicity and Antiproliferative Studies

The antiproliferative and cytotoxic activity of b-TF, RB-TF, and RB aqueous solution at different concentrations (12.5–50 μM) were evaluated on SK-MEL28 melanoma cells; the HFFF2 human fetal foreskin cell line was tested as a control on normal cells. Cells were cultured in Dulbecco’s modified Eagle’s medium (DMEM) (Sigma-Aldrich D5671) supplemented with 10% fetal bovine serum (Euroclone ECS0180L), 2 mM l-glutamine (VWR X0550), 1 mM sodium pyruvate (Sigma-Aldrich S8636), nonessential amino acids (Sigma-Aldrich M7145), 100 units mL^–1^ penicillin, 10 μg mL^–1^ streptomycin, and 25 ng mL^–1^ amphotericin B (AA, VWR L0010) at 37 °C with 5% of CO_2_. Two thousand cells per well were plated in 384-well microplates and incubated for 24 h at 37 °C with 5% CO_2_; the day after, 5 μL of each formulation was added to samples. After 48 h of treatment, cell death was estimated by measuring green fluorescence intensity, provided by staining dead cells with CellTox Green dye (Promega), and cell viability was evaluated by CellTiter-Glo kit (Promega), following manufacturer’s instructions. A second plate was prepared in parallel to assess cell viability at the time of treatment.

### 2.8 Statistical Analysis

Statistical analysis was done with Graph Pad Prism 8.0 software (GraphPad Software Inc. San Diego, CA). The variance analysis (one-way analysis of variance (ANOVA)) was used to analyze the data, followed by Tukey’s multiple comparison test. Differences were considered significant for *p* < 0.05.

## 3 Results

### 3.1 Preparation and Characterization of TF

**Figure 4:**
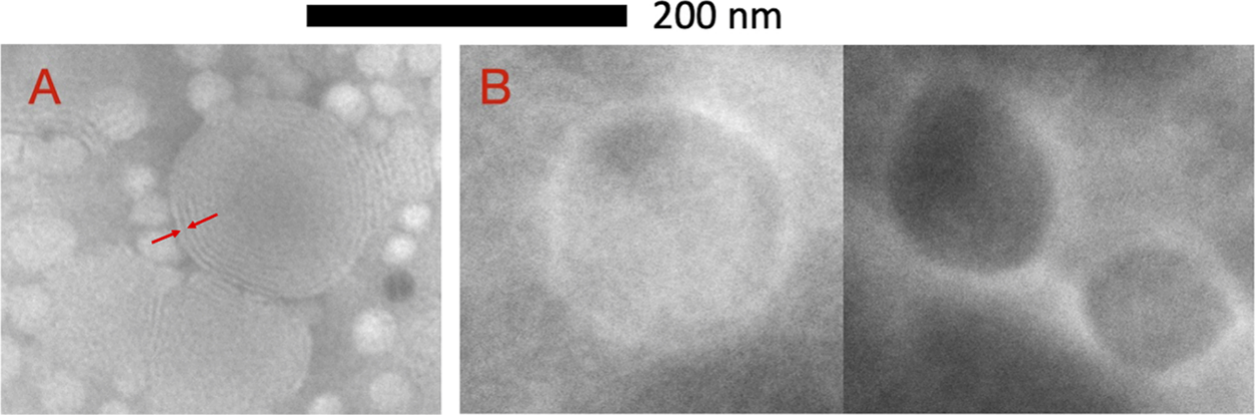
TEM images of b-TF (A) and RB-TF (B). Scale bar: 200 nm; magnification ×30k (A) and ×40k (B). Red arrows show the thickness of the lipid double layer (A).

**Figure 5:**
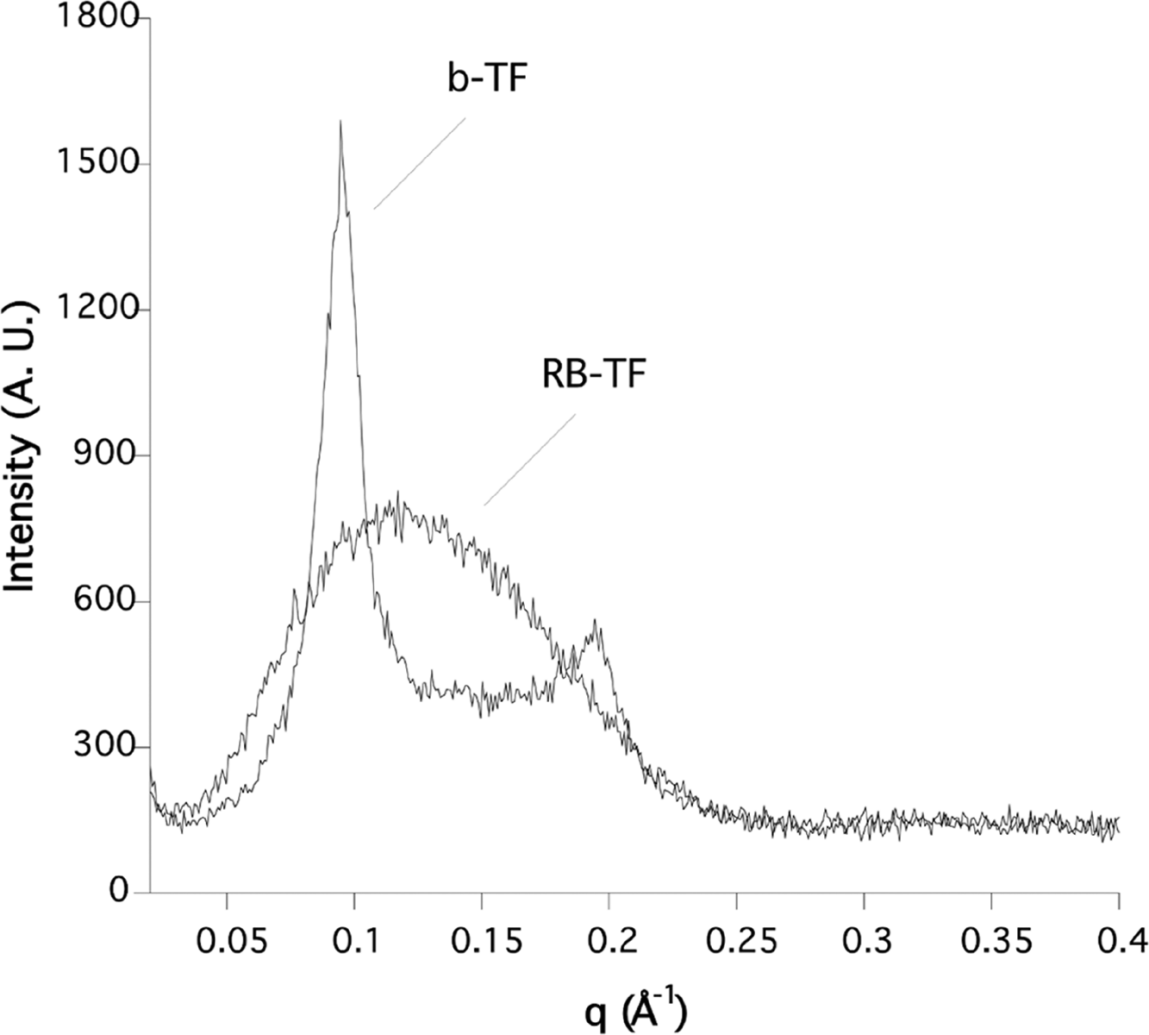
SAXS diffractograms of b-TF and RB-TF.

The TF dispersion was milky, white-colored in the case of b-TF, and pink-colored for RB-TF dispersion; no phase separation or aggregation phenomena were observed. The particle size and PDI (Table 1) indicate that b-TF were in a nanosize range and homogenous (PDI < 0.4); also, b-TF were spherical and multilamellar as seen through TEM (Figure 4). RB-TF showed a PDI similar to b-TF one (*p* > 0.05), proving that encapsulating RB did not perturb the system’s dimensional homogeneity. On the other hand, a slight increase in the particle size was observed compared to b-TF (*p* < 0.01). In addition, nanovesicles surface charge expressed as ζ-potential measurements decreased from a slightly negative value to a highly negative one (*p* < 0.0001) following RB loading (Table 1). TEM images (Figure 4) revealed a morphological difference between the two formulations, as b-TF were multilamellar vesicles (MLV) whereas RB-TF were spherical large unilamellar vesicles (LUV). Also, the SAXS experiments evidenced the differences in terms of lamellarity between the two formulations. Indeed, the diffractogram related to b-TF (Figure 5) revealed the presence of two strong Bragg reflections superimposed to a barely visible diffusive scattering. While the latter was expected when nanoparticles dispersion was analyzed, the observed Bragg peaks proved, beyond any doubt, the high lamellarity of the vesicles. The average inter-bilayer distance (*d*_av_) of 6 nm was calculated from the Bragg relation *d*_av_ = 2π*h*/*q*, where *h* is the Miller index and *q* is the position of the Bragg peaks. On the other hand, the SAXS profile of RB-TF was characterized by a simple diffusive scattering, indicating the absence of lamellarity. To get deeper insights into the TF nanostructure, the double-layer thickness (*d*_B_) was measured in both b-TF and RB-TF. In the case of b-TF, dB (indicated by arrows in Figure 4A) was measured exploiting the TEM images using the ImageJ program and it was found equal to 50 Å. Unfortunately, the same analysis cannot be performed on RB-TF due to the low resolution (see Figure 4B). Therefore, in that case, *d*_B_ was extracted by fitting the SAXS diffractogram using the Global Analysis Program (GAP, see Section 2). The GAP analysis yielded *Z*_H_ = 18 Å. Therefore, a double-layer thickness of 48 Å was calculated, in good agreement with that measured in b-TF liposomes and other similar nanoparticles reported in the literature.^31^ This finding evidenced that the addition of RB to the formulation did not significantly modify the transfersome double-layer thickness, although an evolution from MLV to LUV was observed.

**Table 1:** Characteristics of b-TF and RB-TF^a^

formulation	particle size (nm)	PDI	ζ-potential (mV)	loading efficiency (%)	deformability index
**b-TF**	193.10 ± 7.53**	0.175 ± 0.004	–4.9 ± 0.9***		not deformable
**RB-TF**	206.42 ± 2.67	0.200 ± 0.024	–45.90 ± 0.85	94.50 ± 3.20	1.92 ± 0.06

a Results are expressed as mean value ± standard deviation (*n* = 6); deformability index *n* = 3. **p value < 0.01 vs RB-TF; ***p value < 0.0001 vs RB-TF.

Particle size and PDI of RB-TF did not change after the deformability test (*p* > 0.05) (size: 195.70 ± 0.95 vs 196.07 ± 0.31; PDI: 0.375 ± 0.002 vs 0.387 ± 0.011), whereas the size of b-TF significantly decreased, revealing no deformable vesicles (*p* < 0.01) (size: 226.67 ± 2.80 vs 217.20 ± 2.51; PDI: 0.215 ± 0.012 vs 0.239 ± 0.016). Although the membrane pore size employed in the deformability test did not vary much with the particle size of the tested formulation, RB-TF revealed elastic properties since no change in the dimensional profile was experienced, contrary to the unloaded formulation.

### 3.2 Evaluation of the Interaction between RB and Lipid Phase

**Figure 6:**
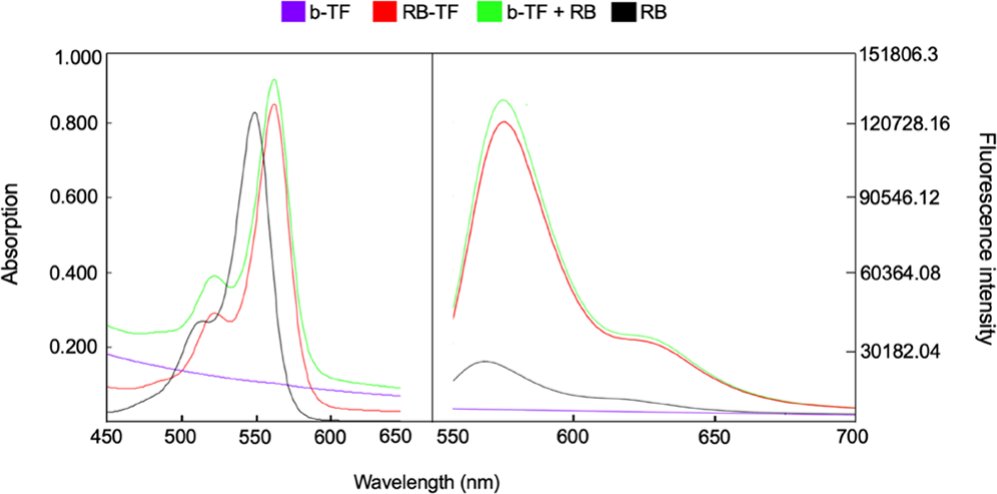
Absorption and emission spectra of RB (7.5 mg L^–1^) included in different molecular environments. RB aqueous solution (black line), b-TF (purple line), RB added to b-TF (green line), and RB-TF (red line).

Two analysis techniques were used to evaluate RB’s ability to interact with lipid nanovesicles, including spectrophotometry and fluorimetry. Figure 6 reports Abs and Ems spectra of RB in the 450–700 nm range in distinct molecular environments.

The spectra demonstrated that RB Abs and Ems λ_max_ are related to the composition of the environment: 549 nm (Abs) and 568 nm (Ems) for RB aqueous solution; 562 (Abs) and 582 (Ems) for both RB added to b-TF and RB-TF; and no peak in Abs or Ems was recorded for b-TF. Both RB Abs λ_max_ and Ems λ_max_ exhibited a shift to a longer wavelength (redshift or bathochromic) when forced to interact with TF. The Abs spectra displayed a slight increase in the RB maximum absorption peak intensity depending on the formulation. In the Ems case, this increase is evident comparing the RB aqueous solution to RB-TF and RB added to b-TF.

### 3.3 Stability Study

**Figure 7:**
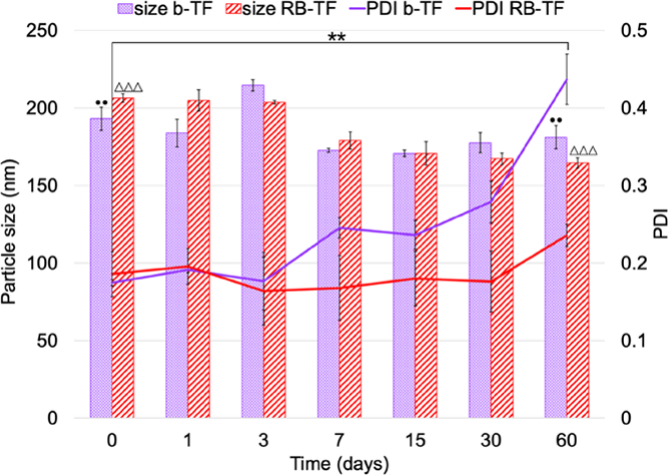
Storage stability. Particle sizes and PDIs of b-TF and RB-TF following storage at 4 °C over 60 days. Results represent the average value of six independent measurements for each formulation (*n* = 6). PDI (**) and particle size (••) of b-TF at 0 day vs 60 days: *p* value < 0.01; particle size of RB-TF at 0 day vs 60 days: *p* value < 0.0001(ΔΔΔ).

The diameter and PDI of TF following storage at 4 °C over 60 days are illustrated in Figure 7.

Encapsulating RB in TF affected the mean particle size and PDI of the system over time. The PDI of b-TF increased from 0.175 ± 0.003 nm at the moment of preparation to 0.437 ± 0.032 nm after 60 days (*p* < 0.01) in a progressive manner; the PDI of RB-TF proved to be reasonably constant during the first 4 weeks, and an irrelevant increase was observed at 60 days, reaching 0.235 ± 0.015 (*p* > 0.05). Both formulations displayed a reduction in the particle size during storage time: b-TF decreased to 181.08 ± 7.40 nm (*p* < 0.01) and RB-TF to 164.55 ± 3.25 nm (*p* < 0.0001). For RB-TF, parallel to changes in the dimensional profile, a reduction of ζ-potential was observed: values decreased from −45.2 ± 1.4 to −35.6 ± 1.6 mV at 60 days (*p* < 0.0001).

The chemical stability test was performed to determine if any interaction between RB and the components of the formulation could occur over time, leading to a degradation of the drug itself. The RB content over time did not vary for both RB aqueous solution and RB-TF dispersion (*p* > 0.05), concluding that no undesired loss of RB occurred and that RB-TF is chemically stable.

**Figure 8:**
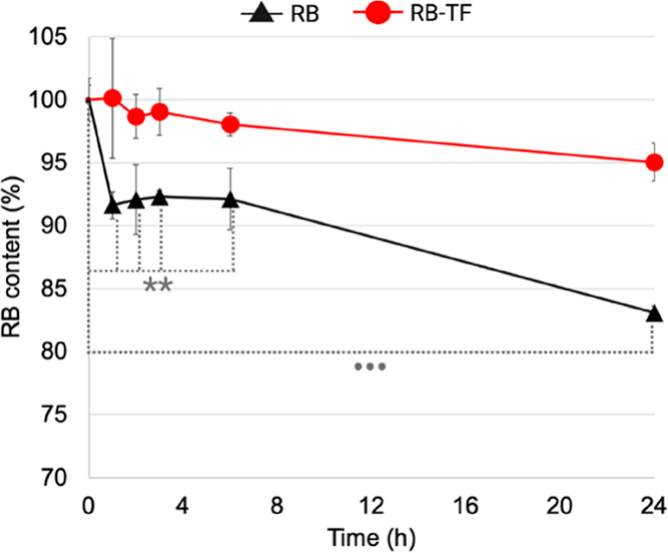
Photostability study. The residual RB in RB aqueous solution and RB-TF dispersion after 24 h of irradiation with visible light. The results represent the mean values of three independent experiments for each formulation (*n* = 3). Initial free RB amount vs free RB amount at 1, 2, 3, and 6 h: *p* value < 0.01(**); initial free RB amount vs free RB amount at 24 h: *p* value < 0.0001 (•••).

Figure 8 reports the residual amount of RB in the RB aqueous solution and RB-TF over 24 h of irradiation under visible light.

Photostability measurements confirmed that the RB aqueous solution undergoes photodegradation: after 1 h, the RB content decreased to 91.63 ± 4.75% (*p* < 0.01); at 6 h, the RB content was 92.11 ± 0.9% (*p* < 0.01) and reached 83.09 ± 1.49% at the end of 24 h (*p* < 0.0001). On the other hand, RB-TF dispersion efficiently protected RB from visible light over 24 h: at the end of the experiment, the RB content in RB-TF was reported to be 95.05 ± 1.49% (*p* > 0.05).

### 3.4 In Vitro Release Study

**Figure 9:**
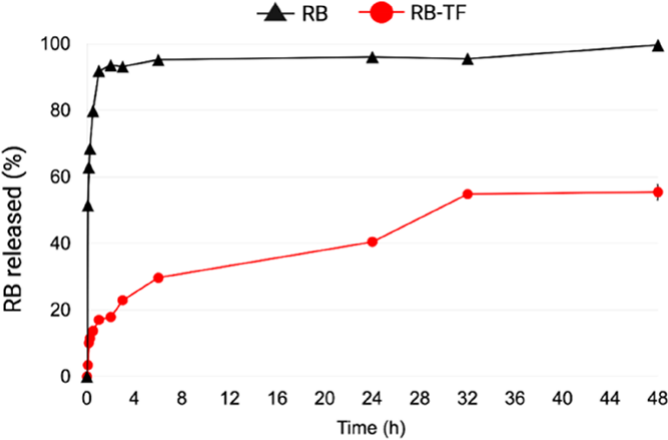
In vitro release profiles of free RB and RB-TF dispersion. The cumulative amount of RB aqueous solution (black line) and RB-TF (red line) recovered in the acceptor medium over 48 h (*n* = 3).

Figure 9 depicts the in vitro release profile of RB-TF dispersion and the cumulative amount of RB aqueous solution in the acceptor medium. The results represent the mean values of three independent experiments for each formulation over 48 h, following a single dose of RB aqueous solution and RB-TF dispersion (RB = 1 mg).

As data proved, when RB aqueous solution was tested, 51.43 ± 0.15% of the dye was detected within 5 min, and 91.82 ± 0.3% in 1 h, reaching 99.60 ± 0.3% at the end of the experiment. On the contrary, the RB-TF dispersion release rate was slower than that of RB aqueous solution: the maximum RB amount released was 54.81 ± 0.25%, reached after 32 h.

The lack of a burst RB release for RB-TF dispersion suggests that a significant amount of RB interacted with lipid nanovesicles, and thus, it is not in solution such as in RB aqueous solution.

### 3.5 Ex Vivo Epidermis Permeation and Retention Study

**Figure 10:**
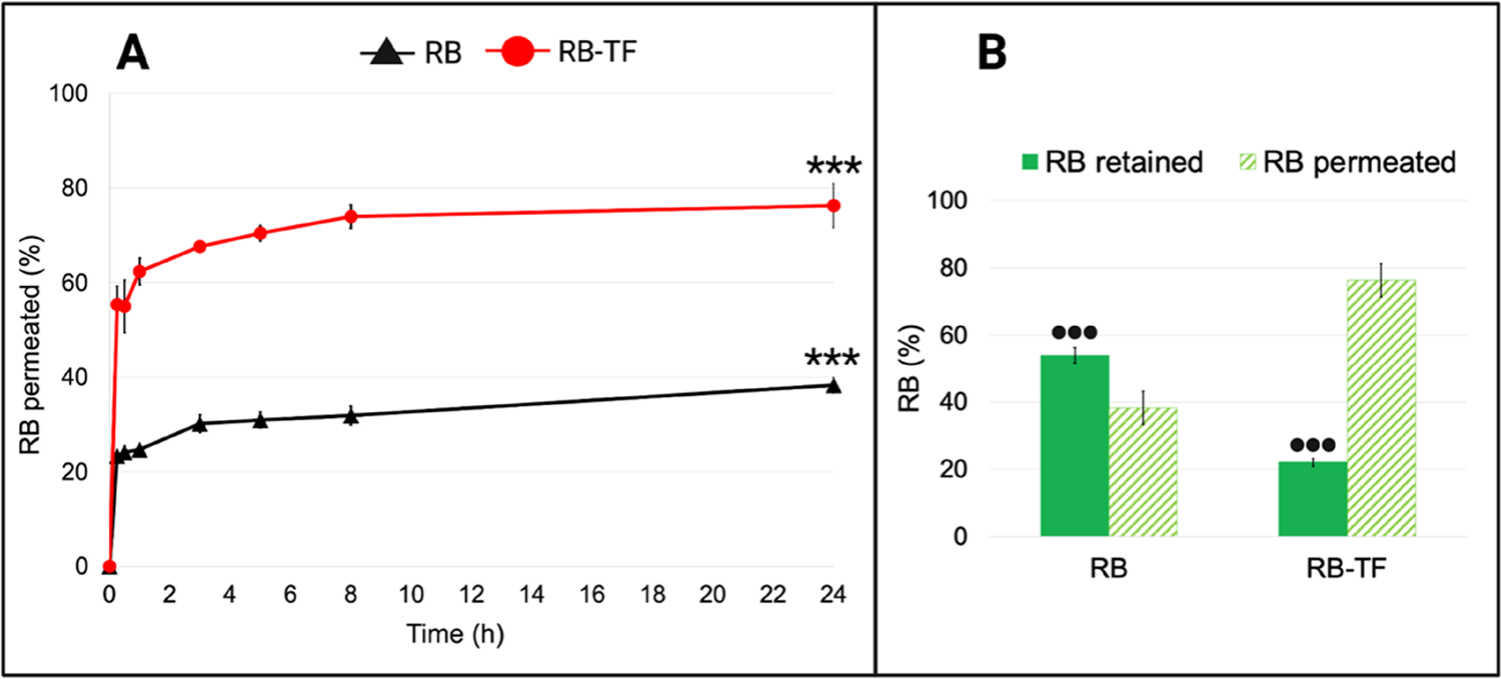
Ex vivo epidermis permeation and retention profiles of RB aqueous solution and RB-TF dispersion. (A) The cumulative amount of RB aqueous solution (black line) and RB-TF dispersion (red line) permeated through the epidermis over 24 h (*n* = 3). RB-TF dispersion permeated after 24 h vs RB aqueous solution permeated after 24 h: *p* value < 0.0001(***). (B) Amount of RB aqueous solution and RB-TF dispersion retained by the epidermis (full green bar) and totally permeated (striped bar) at the end of the ex vivo permeation test (*n* = 3). RB-TF retained by the epidermis and RB aqueous solution retained by the epidermis: *p* < 0.0001(•••).

The ex vivo permeation and retention studies were carried out to predict the delivery performance of RB-TF dispersion across the epidermis compared to RB aqueous solution. Figure 10 reports the average values of RB permeated through the epidermis and RB retained over 24 h, following a single dose of RB aqueous solution and RB-TF dispersion (RB = 0.5 mg). The results represent the average of three independent experiments for each formulation.

The amount of RB aqueous solution permeated through the epidermis was 24.77 ± 0.88% after 1 h, and it increased to 38.31% at the end of 24 h. On the other hand, RB-TF dispersion was found to permeate by 55.40 ± 3.93% within 15 min, reaching its maximum after 8 h (73.99 ± 2.49%). At 24 h, an irrelevant increase of RB-TF dispersion permeation was observed (*p* > 0.05). In parallel, the drug amount detected in the epidermal layer after 24 h was significantly different for RB aqueous solution and RB-TF dispersion (*p* < 0.0001): 54.00 ± 2.37% of RB aqueous solution was retained by the skin, whereas RB-TF dispersion allowed decreasing RB retention to 22.08 ± 1.14%. An ex vivo permeation study proved that RB-TF dispersion doubled RB’s amount that permeates the epidermis in 24 h, limiting the skin deposition phenomena.

### 3.6 Cytotoxicity and Antiproliferative Studies

**Figure 11:**
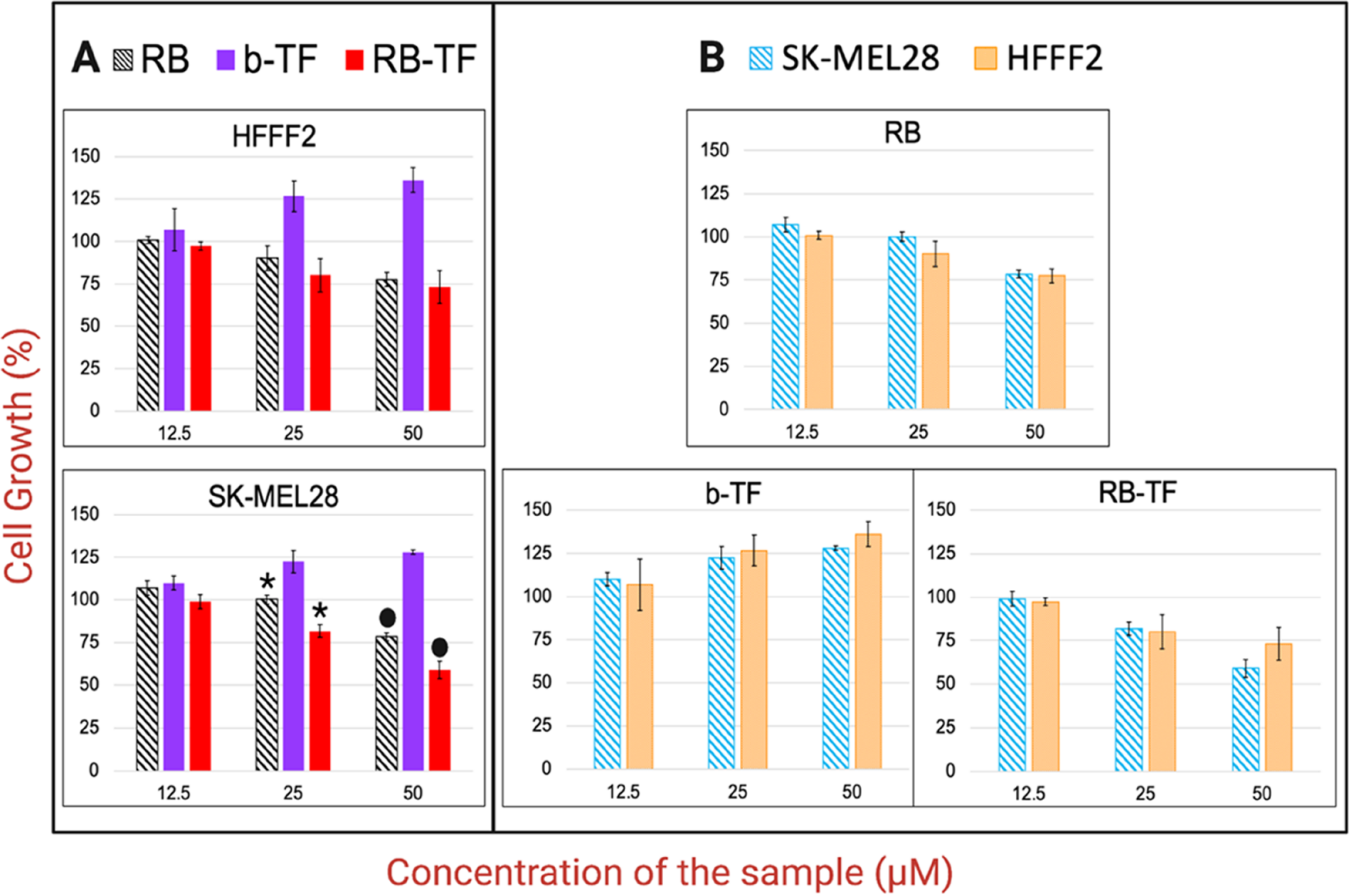
Effects of RB aqueous solution, RB-TF, and b-TF on the SK-MEL28 and HFFF2 cell growth. Cells were cultured with samples for 48 h. (A) The viability of each cell line depending on the formulation and the concentration tested. (B) Analysis of each formulation’s impact at different concentrations, comparing the growth suppression of the two cell lines. The effect of RB aqueous solution (25 μM) on SK-MEL28 vs the effect of RB-TF (25 μM) on SK-MEL28: *p* value < 0.05 (*). The effect of RB aqueous solution (50 μM) on SK-MEL28 vs the effect of RB-TF (50 μM) on SK-MEL28: *p* value < 0.05 (•).

The impact of RB aqueous solution and RB-TF on cell death and viability was evaluated on SK-MEL28 and HFFF2 cells (Figure 11). After 48 h, no cell death induced by the formulations was observed (data not shown). Both cell lines displayed a dose-dependent reduction of growth response to RB aqueous solution and RB-TF. The data suggested that at both concentrations of 25 and 50 μM, the RB-TF cell growth reduction was significantly higher than RB aqueous solution (*p* < 0.05). At a concentration of 50 μM, RB aqueous solution inhibited the percentage of growth of SK-MEL 28 and HFFF2 without selectivity (*p* > 0.05); on the other hand, RB-TF displayed a potential selectivity for SK-MEL28 at this concentration (*p* = 0.05). On the contrary, no growth reduction was noticed in b-TF, which instead promoted cell growth.

## 4 Discussion

The current work aimed to determine if a proper delivery system can enhance the antimelanoma intrinsic activity of Rose Bengal. Considering the localization of primary melanoma tumor, the dermal administration was here investigated as it represents the most direct and noninvasive access route to cancer cells, obviating the need for intralesional injection. We developed deformable lipid nanovesicles to vehicle RB across the epidermis and to reach the dermal layer. In this way, RB-TF could potentially eradicate melanoma lesions at early stages localized in the outermost layer of the skin (melanoma in situ) and localized but invasive melanoma, which advances deeper in the dermis.^32^

Based on the high water solubility of RB disodium salt, TF were prepared using the modified reverse-phase evaporation (REV) technique.^20^ This method allows obtaining vesicles with a high aqueous space-to-lipid ratio and, consequently, to entrap a large percentage of drug solubilized in the aqueous phase.^33^ The original REV technique, first reported in 1978 by Szoka et al.,^34^ was used to study several phospholipids, either pure or mixed with other lipids, mainly involving diethyl ether, isopropyl ether, and chloroform as solvents. Herein, the lipid phase was made of phosphatidylcholine, cholesterol, and Span 80 as the surfactant. Cholesterol was added to improve liposomes’ resistance to leakage and degradation in vivo, as it is known to provide rigidity to the structure;^35^ simultaneously, the presence of the surfactant destabilizes the lipid bilayer, increasing the vesicle’s deformability. As previously reported by Joshi et al.,^36^ a suitable balance of cholesterol and surfactant can provide the desired flexibility to the lipid vesicle to improve the migration toward the deepest region of the skin. Moreover, the surfactant can improve properties such as encapsulation efficiency, stability, and permeability.^15,37^ In addition, ethanol has been employed to replace solvents originally used (Supporting Information) to obtain a formulation as safest as possible. Ethanol is a solvent with low toxic potential (*Pharmacopoeia Italica*, XII ed.), whereas the toxicity of ethers and halogenated hydrocarbons is well known.^38^ To optimize the dimensional properties of nanoparticles, formulations were finally sonicated and extruded (Supporting Information). Probe sonication reduced the particle size, as previously reported by Zahra Hadian Sahari et al.,^39^ who evaluated the effect of sonication on similar lipid vesicles. Since lipid components here used are heat-sensitive, the formulations were sonicated in cycles to permit TF to cool and avoid thermal degradation.^21^ The extrusion process allowed a narrow particle size distribution because it forced the dispersion to pass several times through a membrane with uniform pores.^25^

The final TF dispersions appeared turbid and milky, indicating that the average particle size was above 100 nm; indeed, formulations having an average particle size of 100 nm or smaller are instead characterized by a transparent, bluish appearance (Tyndall effect).^40^ PCS later confirmed dimensional properties: b-TF and RB-TF had a particle size of around 200 nm. Such a result agrees with the study conducted by Godbole et al.,^41^ reporting that a milky appearance characterizes liposomes above 300 nm and those ranging between 200 and 300 nm. Also, even if the average size of formulations was around 200 nm and a PDI below 0.2, such values do not exclude the presence of vesicles larger than 200 nm, affecting the milky appearance of the dispersion.

Lipid vesicles whose size was between 100 and 600 nm proved to enhance skin penetration of water-soluble photosensitizer drugs and, in particular, TF ≤ 300 nm reached deeper into the skin.^13,15^

ζ-Potential is a fundamental parameter to predict the system’s stability, as it measures the magnitude of the repulsion between charged particles in the dispersion. The higher ζ-potential value denotes highly charged particles and minimizes the possibility of aggregation via electrostatic interaction. Conventionally, a sample exhibiting a ζ-potential value higher than |30| mV is considered stable.^25^ The ζ-potential measurement showed that TF’s surface charge significantly decreased following RB loading, reaching −45.9 mV. As previously observed for indocyanine green zein-phosphatidylcholine nanoparticles, this suggests that the negatively charged RB was incorporated into the phospholipid bilayers and the incorporation improved the electrostatic stabilization of the system.^42^ Such a result agrees with the storage stability observed for b-TF and RB-TF. The PDI value of b-TF increased from the preparation. In contrast, the PDI of RB-TF remained relatively constant, implying that b-TF progressively aggregates over time and that the RB presence limits the aggregation phenomena.

The stability of RB-TF was also studied in terms of drug content over time and photostability. Over time, no reduction in the RB content was noticed, evidencing the chemical compatibility between the formulation and the drug components. On the contrary, we observed that photodegradation of RB aqueous solution was significantly faster than RB-TF dispersion and that the latter efficiently protected RB over 24 h. As already observed by Ali et al.,^43^ this difference can be related to the light scattering exerted by lipid, reducing the photon energy delivered to RB associated with vesicles. Because the dermal administration is an administration route exposed to visible light, protecting RB is essential to avoid an undesirable drug loss.

Morphological investigation (TEM) revealed MLV b-TF and LUV RB-TF, as also proved by SAXS analysis. The formation of LUV is a consequence of the RB presence, which is likely to intercalate in the hydrophobic palisade of the phospholipid bilayer, thus modifying the effective lipid packing parameter *P* = *v*/*a*_0_*l*_c_, where *v* represents the volume of the hydrophobic chain, *a*_0_ is the headgroup area, and *l*_c_ is the critical hydrocarbon chain length (approximately equal to the fully extended chain length).^44,45^ Such alteration of *P* can be undoubtedly called into play to explain the different lamellarity degrees observed in b-TF and RB-TF and may also play a role in the different behaviors of nanovesicles in terms of deformability. It has been previously discussed that cholesterol provides rigidity to the liposomal structure; only a suitable balance of cholesterol and surfactant can provide the desired flexibility leading to a deformable formulation. Dudhipala et al.^46^ observed that the optimal concentration of Span 80 to obtain the best deformability index for aceclofenac-transferosomes is 0.15% w/v and that higher concentrations reduce the deformability. The authors hypothesized that this is due to the linear structure of Span 80, which tends to compact the double layer and, therefore, reduce the TF’s deformability. This evidence and the presence of cholesterol can explain the nondeformability of b-TF. On the other hand, Bouvrais et al.^47^ proved that the bending rigidity of lipid membranes strongly depends on the organization of lipid bilayers, which can be modified by additives or environmental modification, e.g., by introducing salt and buffers. In agreement with the deformability indexes observed, it can be assumed that adding RB to the lipid bilayer induced a softening effect on b-TF, resulting in unilamellar and deformable RB-TF.

The spectrophotometric and fluorimetric analyses further confirmed the interaction between RB and lipid components. For RB-TF dispersion and RB added to b-TF dispersion, the RB absorption λ_max_ shifted to a higher wavelength compared to RB aqueous solution. Since the dye’s solvatochromic behavior has been reported, λ_max_ is indicative of the microenvironment interacting with RB when most of the dye is bound to the dispersed phase.^48^ The redshift observed in the absorption spectra implies that RB sensed the less polar environment of TF and migrated. Also, the emission spectra of RB-TF dispersion showed an increase in the fluorescence signal. As previously reported by Chang et al.,^27^ the higher intensity ratio suggests that free RB intercalated through the lipid bilayer mainly as a monomeric form but not as a dimer form, effectively limiting RB aggregates’ formation. Nevertheless, the logarithm of the partition coefficient (log *P*) of the RB disodium salt is reported to be 0.59, resulting in an amphiphilic molecule.^6^ Considering the amphiphilic properties and high water solubility, the distribution of RB both in the external lipid bilayer and in the internal aqueous core of the TF is expected.

The above characterization agrees with the in vitro release and ex vivo permeation profiles obtained experimentally. The in vitro release assay proved that RB aqueous solution quickly diffuses through the hydrophilic 50 nm-size polycarbonate membranes, whereas RB-TF dispersion released RB slower and in a controlled manner. In contrast, the ability of the RB aqueous solution to permeate the epidermis and consequently reach the dermis was limited by RB’s physical–chemical profile. The epidermis is the outermost layer of the skin; it comprises different living epithelial cell strata (viable epidermis) localized beneath a multilayer of dead cells called corneocytes (stratum corneum, SC). As previously stated in the Introduction section, SC is considered the main barrier against the penetration of external agents,^11^ significantly when molecules exceed 500 Da and present anionic or cationic charges.^12,15^ RB is an amphiphilic drug but it presents two anionic charges and a molecular weight of 1017.64 g mol^–1^.^6^ As proved by the ex vivo skin permeation study, less than 40% of the RB aqueous solution permeated through the epidermis and most of it was retained by the epidermal layer. Formulating RB in TF doubled the RB amount that permeated the epidermis and effectively reached the dermal layer. Herein, no studies evaluating the mechanism of RB-TF’s permeation were performed. However, due to their deformability, the intact permeation of nanovesicles through the skin is supposed to be the primary operating action. In support of this hypothesis, the elastic properties of RB-TF were assessed and previously discussed. Nevertheless, other mechanisms of action cannot be excluded; these include the structural modification of the intracellular lipid matrix due to the penetration enhancing effect of the surfactant and the vesicles adsorption to and/or fusion with the stratum corneum.^18,19^

The cytotoxicity of RB and RB-TF in SK-MEL28 cells was determined after 48 h of exposure. Although RB is mainly considered for its photodynamic inactivation of cancer and microbial cells, previous evaluations of its intrinsic cytotoxicity were reported. It was observed that RB concentrations ranging between 5 and 10 μM induce cell death only following RB photosensitization. The intrinsic toxicity of RB on melanoma and breast cancer cell lines was observed at a concentration of 100 μM; in particular, it was evident for melanoma in the case of 200 μM and for breast cancer at 300 μM.^8,49^ Such results agree with the values obtained in SK-MEL28 cells. Herein, we evaluated RB-induced toxicity up to a maximum of 50 μM in the absence of external stimuli, including light or ultrasounds. At this concentration, a significant cytostatic effect was observed. Based on this outcome, we also investigated the efficacy of RB-TF to reduce the viability of SK-MEL28. The cytostatic effect of RB-TF was higher than free RB, and the explanation is based on the chemical profile of RB. RB is an amphiphilic water-soluble compound with a more hydrophilic tendency due to two negative charges at physiological pH; these characteristics make it difficult for RB to cross lipophilic cell membranes spontaneously, resulting in an insufficient cell accumulation at low concentrations in the absence of a carrier. We hypothesized that the association of RB with the lipophilic TF increased the RB cell uptake and consequently the cytostatic effect.^6^ On the other hand, b-TF did not induce any effect supporting the thesis of a safe nanocarrier enhancing RB antimelanoma activity. In addition, at 50 μM, a promising selectivity of RB-TF on cancer cells compared to fibroblast was noticed.

## 5 Conclusions

We have developed RB-loaded transfersomes to combine the permeation ability of deformable lipid vesicles with the dye’s intrinsic antimelanoma activity. The preparation technique employed (REV) avoided using toxic organic solvents and allowed one to obtain monodispersed nanoparticles in a dimensional range suitable for dermal delivery; RB efficiently associated with the lipid carrier. Ex vivo epidermis permeation confirmed that TF significantly improved the permeation of RB across the epidermal barrier. Furthermore, in vitro antiproliferative assays indicated a higher intrinsic cytostatic effect of RB-TF compared to free RB. Considering these outcomes, RB-TF represent a promising approach to fight against primary cutaneous melanoma without involving sono-photodynamic treatments. Future studies will involve the development of a RB-TF controlled release dosage form and the evaluation of dermatokinetic vs its pharmacokinetic when administered intravenously.

## Supporting Information Available

Preformulation study of transfersomes; and illustration of the dissolution apparatus employed in the in vitro release study (PDF)

## Notes

Graphical abstract, Figures 1–3, andS2 reported in the Supporting Information were created with BioRender.com.

## Abbreviations

AIRC: Italian Association for Cancer Research
RB: Rose Bengal
SC: stratum corneum
TF: transfersomes
RB-TF: Rose Bengal-loaded transfersomes as dispersion
REV: reverse-phase evaporation
US: ultrasound
b-TF: unloaded transfersomes as dispersion
PCS: photon correlation spectroscopy
PDI: polydispersity index
DC (%): drug content percentage
TEM: transmission electron microscopy
SAXS: small-angle X-ray scattering
MLV: multilamellar vesicles
LUV: large unilamellar vesicles
Abs: absorbance
Ems: emission
λ_max_: maximum wavelength (nm)
GAP: Global Analysis Program
