# Supplementary material for: Improving Dermal Delivery of Rose Bengal by Deformable Lipid Nanovesicles for Topical Treatment of Melanoma
Source: Mol Pharm. 2021 Sep 23;18(11):4046–57. doi: 10.1021/acs.molpharmaceut.1c00468 (PMC8564756; doi:10.1021/acs.molpharmaceut.1c00468)
Supplement: Supplementary file 1 — mp1c00468_si_001.pdf [file mp1c00468_si_001.pdf]

Supporting Information

Improving dermal delivery of Rose Bengal by deformable lipid nanovesicles for melanoma topical treatment

Sara Demartis[1]; Giovanna Rassu[1]; Sergio Murgia[2,3]; Luca Casula[2]; Paolo Giunchedi[1]; Elisabetta Gavini*[1]

[1] Department of Chemistry and Pharmacy, University of Sassari, Italy.

[2] Department of Life and Environmental Sciences, University of Cagliari, Italy.

[3] CSGI, Consorzio Interuniversitario per lo Sviluppo dei Sistemi a Grande Interfase, Via della Lastruccia 3, I-50019 Sesto Fiorentino, Florence, Italy.

*Correspondence: eligav@uniss.it; Tel.: +39-079 228 752

Preformulation study of deformable lipid vesicles.

1. Materials. Rose Bengal sodium salt (RB), cholesterol, Span® 80, chloroform, ethanol, methanol and dyethil ether were purchased by Sigma-Aldrich (St. Louis, MO). Lipoid S 100 was gifted by Lipoid GmbH (Ludwigshafen, Germany). Ultrapure bi-distilled water was obtained by a MilliQ R4 system, Millipore (Milan, Italy).

2. Methods.

2.1. Transfersomes (TF) Preparation. Two series of TF were developed, TF1 and TF2. About TF1, TF1-A and TF1-B were studied (Table 1).TF1-A were prepared by thin-film hydration method (TFH), TF1-B by reverse-phase evaporation method (REV). To obtain TF1-A 100.8 mg of Lipoid S 100, 31.29 mg of cholesterol and 16 µL of Span® 80 were dissolved in a mixture of ethanol, methanol, and chloroform (2:2:1) which was completely evaporated under vacuum (u.v.) at 40 °C. The lipid film formed was hydrated with 20 mL of MilliQ water for 1 hour at 40°C by a rotary evaporator (Rotavapor RE111, Büchi LabortechnikAG, Flawil, Switzerland). The dispersion was kept for 1 hour at r.t. and sonicated testing two techniques (Table 2). TF1-B were prepared dissolving 100 mg of Lipoid S 100, 20 mg of cholesterol and 10 µL of Span® 80 in a mixture of diethyl ether and chloroform (3:1), 10 mL of MilliQ water were added and sonicated for 4 min at 50% US providing an emulsion which was evaporated u.v. at 40 °C until complete solvent evaporation. The gel formed was kept at r.t. overnight to be converted into the transfersomal dispersion which was sonicated testing two sonications (Table 2). Blank TF1 (bTF1-A, bTF1-B) and loaded TF1 (RBTF1-A, RBTF1-B) were prepared considering the outcomes of the preformulative study, loaded TF1 were obtained using 2 µM RB aqueous solution instead of MilliQ water. TF1 were stored for 3 months at 4°C to identify the best preparative technique. The leader formulation (RB-TF) was prepared based on TF1 study changing solvent and sonication time (Table 2). RB-TF were prepared by REV technique: 400 mg of Lipoid S 100, 75 mg of cholesterol and 40 µL of Span® 80 were dissolved in ethanol at 50 °C and mixed with 10 mL of RB aqueous solution (RB=500 µM) sonicated for 30 s at 50% US amplitude and evaporated u.v. at 50 °C until complete solvent evaporation. The dispersion was kept for 1 hour at r.t. and sonicated by 3 sonication cycles: each cycle consisted of 10 s of US followed by an interval of 20 s. RB-TF were extruded 10 times through a regenerated cellulose syringe filter (pore size: 0.45 µm, filter size: 25 mm, AlfaTech, Genova, Italy). Corresponding blank (b-TF) was prepared.

Formulation	Technique	Solvents	Sonication
**TF1-A**	TFH	Ethanol<br>Methanol<br>Chloroform	Probe 50% (60 s, 90 s, 120 s)<br>Bath 40°C (5',10', 15')
**TF1-B**	REV	Diethyl ether<br>Chloroform	Probe 50% (60 s, 90 s, 120 s)<br>Bath 40°C (5',10', 15')
**TF-2**	REV	Ethanol	45% US

Table 1. Preformulative parameters modified during TF preparation.

2.2. Particle size analysis. Dimensional analysis of formulations was performed by photon correlation spectroscopy (PCS). A Coulter nanosizer N5 (Beckman-Coulter Inc., Miami, FL, USA) was used to determine the hydrodynamic diameter and the polydispersity index (PDI) to measure the dimensional heterogeneity of the sample. Before each determination, all samples were diluted adequately with MilliQ water previously filtered (regenerated cellulose syringe filter, pore size: 0.20 µm, filter size: 15mm, Albet LabScience, Dassel, Germany) to ensure light-scattering intensity within the required range of the instrument (between 5×104 and 1×106 counts s−1). Particle size was calculated in unimodal using the following conditions: fluid refractive index 1.333; temperature 25°C; viscosity 0.890 centipoises; angle of measurement 90°; sample time 3.0 ms and sample run time 300 s.

3. Results. Figure 1 shows the effects of sonication on TF1 dimensional profile. Not sonicated TF1-A had a size of 659.03±10.54 nm, TF1-B size was above 1µm. 2 min of probe sonication at 50% US amplitude was selected as sonication method since it allowed to obtain vesicles of 182.47 nm for TF1-A and 206.63 nm for TF1-B. Bath sonication (Bandelin Sonorex RK 52 Heinrichstrable, Berlin, Germany) of TF1-A revealed a good dimensional profile but it was discarded due to the presence of dispersed aggregates. RBTF1-A, RBTF1-B and corresponding blanks were prepared using selected sonication parameters. Storage stability showed that TF1-B was the most stable: RB TF1-A size increased from 214.4±4.38 to over 1µm, RB TF1-B increased from 297.6±1.41 nm to 437.65±4 nm; a similar increase was seen for bTF1.

Considering these outcomes, RB-TF were prepared by REV technique and probe sonication method.

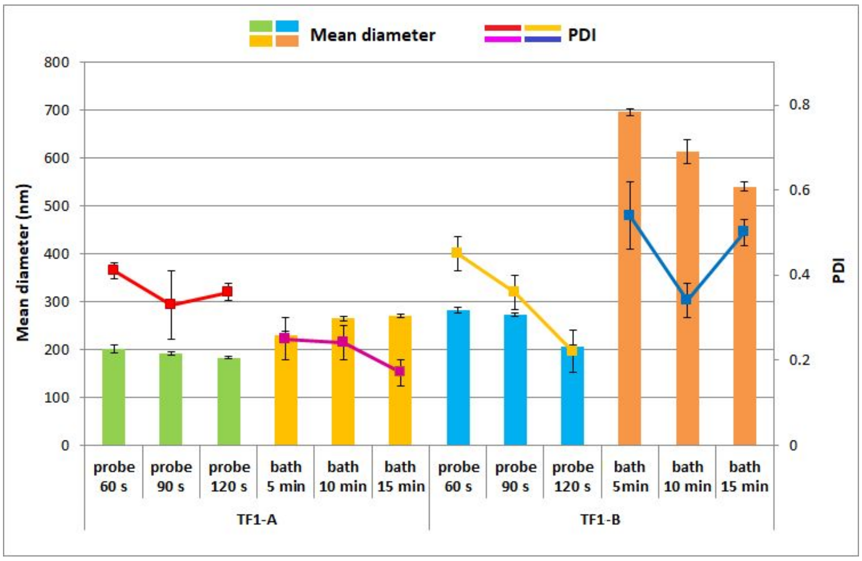

Figure 1S. Influence of sonication technique and time on TF1 dimensional profile.

In vitro release study.

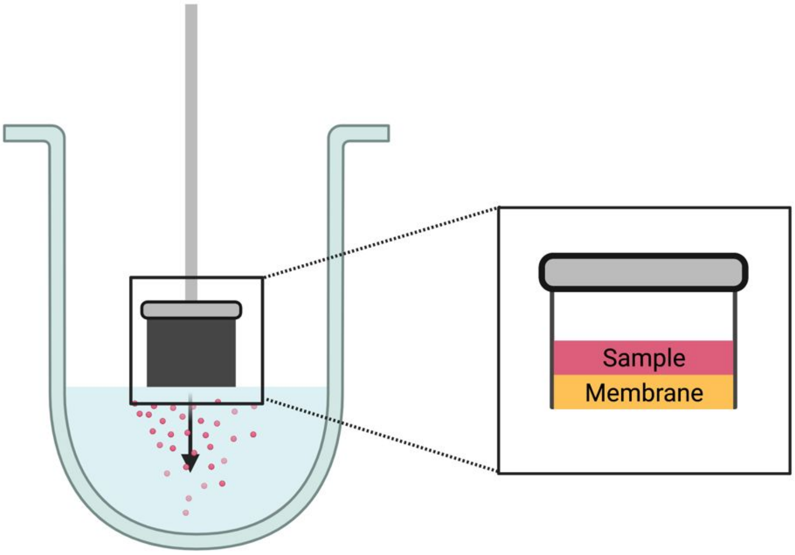

Figure 2S. Schematic illustration of the dissolution apparatus employed to perform the in vitro release study. In vitro release profile of RB-TF dispersion was evaluated across a polycarbonate membrane (0.050 µm pore size, 47 mm diameter, Sigma-Aldrich, St. Louis, MO, USA) using a dissolution apparatus (DT 70, Erweka, Langen, Germany), as previously described by Gavini et al.[28]. The membrane was mounted on the bottom of cylindrical plastic support consisting of a tube (height=1.91 cm, diameter=2.28 cm) connected to a drive shaft of the dissolution apparatus. The membrane was clamped to the support by a plastic ring, and then 2 mL of the sample (1 mg of RB) were placed on the surface of the membrane. The system was then inserted into the vessel

containing 250 mL of PBS as the acceptor medium, keeping the membrane in contact with the surface of the PBS all the time. RB aqueous solution was also tested as comparison.
